# A Socially Assistive Robot for Long-Term Cardiac Rehabilitation in the Real World

**DOI:** 10.3389/fnbot.2021.633248

**Published:** 2021-03-16

**Authors:** Nathalia Céspedes, Bahar Irfan, Emmanuel Senft, Carlos A. Cifuentes, Luisa F. Gutierrez, Mónica Rincon-Roncancio, Tony Belpaeme, Marcela Múnera

**Affiliations:** ^1^Biomedical Engineering Department, Colombian School of Engineering Julio Garavito, Bogotá, Colombia; ^2^Centre for Robotics and Neural Systems, University of Plymouth, Plymouth, United Kingdom; ^3^Department of Computer Sciences, University of Wisconsin-Madison, Madison, WI, United States; ^4^Fundación Cardioinfantil-Instituto de Cardiología, Bogotá, Colombia; ^5^IDLab-imec, Ghent University, Ghent, Belgium

**Keywords:** social assistive robotics, cardiac rehabilitation, human-robot interaction, long-term interaction, social robot, human-robot interface

## Abstract

*What are the benefits of using a socially assistive robot for long-term cardiac rehabilitation*? To answer this question we designed and conducted a real-world long-term study, in collaboration with medical specialists, at the Fundación Cardioinfantil-Instituto de Cardiología clinic (Bogotá, Colombia) lasting 2.5 years. The study took place within the context of the outpatient phase of patients' cardiac rehabilitation programme and aimed to compare the patients' progress and adherence in the conventional cardiac rehabilitation programme (*control condition*) against rehabilitation supported by a fully autonomous socially assistive robot which continuously monitored the patients during exercise to provide immediate feedback and motivation based on sensory measures (*robot condition*). The explicit aim of the social robot is to improve patient motivation and increase adherence to the programme to ensure a complete recovery. We recruited 15 patients per condition. The cardiac rehabilitation programme was designed to last 36 sessions (18 weeks) per patient. The findings suggest that robot increases adherence (by 13.3%) and leads to faster completion of the programme. In addition, the patients assisted by the robot had more rapid improvement in their recovery heart rate, better physical activity performance and a higher improvement in cardiovascular functioning, which indicate a successful cardiac rehabilitation programme performance. Moreover, the medical staff and the patients acknowledged that the robot improved the patient motivation and adherence to the programme, supporting its potential in addressing the major challenges in rehabilitation programmes.

## 1. Introduction

Cardiovascular diseases (CVDs) are a group of disorders of the heart and blood vessels that include cerebrovascular diseases, rheumatic heart diseases and other conditions (World Health Organization, 2011). CVDs are the cause of 17.7 million deaths every year, approximately 31% of all deaths worldwide (World Health Organization, 2011). Within the CVDs, ischemic heart disease causes 8.76 million deaths, and strokes cause 6.24 million deaths^1^ worldwide each year.

Cardiac rehabilitation (CR) following a cardiovascular event is a Class I recommendation of the European Society of Cardiology, the American Heart Association, and the American College of Cardiology (Thomas et al., 2007; Piepoli et al., 2010; Galve et al., 2014). A typical CR programme promotes a healthy lifestyle, reduces risk factors, improves health-related quality of life, and decreases mortality and morbidity (Oldridge et al., 1988; Taylor et al., 2004, 2012; Clark et al., 2005; Kraus and Keteyian, 2007; Lawler et al., 2011; Anderson et al., 2016; Giuliano et al., 2017). CR programme is generally conducted in three phases (Kraus and Keteyian, 2007): (I) *inpatient*, (II) *outpatient*, and (III) *maintenance* phase. The inpatient phase starts after the patient is hemodynamically stable, typically after 48 h of the surgery procedure. The patient performs low-intensity movements for maintaining muscle tone and reducing risks or any complication at this phase. The outpatient phase starts after the patient is discharged, and lasts on average 18 weeks with sessions twice per week. During this phase, the patient performs physical exercises at the hospital, and receives an educational programme about the risk factors to gain healthy habits (e.g., controlling blood pressure, cholesterol, weight, and stress management). The maintenance phase aims to reinforce the information and habits gained during the outpatient phase and lasts on average about 9 months with one or two sessions per week. The physical exercises in the outpatient phase typically last 1 h, and consist of (1) *warm-up* via stretching exercises, (2) *training* by physical exercises, e.g., on a treadmill, and (3) *cooldown* during which low-intensity exercises are carried out. In a conventional CR session, during *warm-up* and *cooldown*, the medical staff measures the initial and final heart rate and blood pressure. During *training*, the heart rate and the exertion level of the patient are requested by the medical staff regularly to determine whether there is a need for the intervention to decrease the intensity of the exercise, which is determined by the speed and inclination of the treadmill. Our work focuses on the *training* step to provide individual and immediate feedback during the workout, and alert the medical staff in the case of critical biomedical values.

Adherence to the CR programme is vital for the complete recovery of a patient and to reduce the risk of suffering recurrent events (Jolly et al., 2007; Suaya et al., 2009; Hammill et al., 2010). Nonetheless, in addition to the low participation in the programme (Altenhoener et al., 2005; McKee et al., 2014), a high percentage (24–50%) of patients who enroll in cardiac rehabilitation programmes drop out (Carlson et al., 2000; Scane et al., 2012). The reasons behind dropout or non-attendance vary, such as motivation factors, lack of interest or faith in the CR programme, increasing lack of motivation throughout the CR programme (Siegert and Taylor, 2004; McKee et al., 2014), anxiety about the exercise component (Cooper et al., 2007), a lack of group cohesion (Maclean and Pound, 2000; Beswick et al., 2005), presence of comorbidities (e.g., depression, obesity, diabetes) (Turk-Adawi et al., 2013), poor funding or poor organization (Bethell et al., 2009), difficulties with the location or accessibility (Turk-Adawi et al., 2013), scheduling or work commitments (Ruano-Ravina et al., 2016), and negative beliefs (Shahsavari et al., 2012). While other factors are beyond the control of the healthcare staff, motivational issues can be addressed by providing individual support within the sessions, through rigorous supervision during the patient's exercise and quick help in emergent situations (Shahsavari et al., 2012). However, the CR programme at clinics is generally conducted with large groups, and it is challenging for healthcare staff to provide continuous and individual support during the session (Turk-Adawi et al., 2019). In this context, integrating a socially assistive robot (Feil-Seifer and Matarić, 2005) can help provide one-on-one support to the patient, which, in turn, can facilitate the healthcare staff to focus on the individual needs of patients, immediately detect any complications during the session, analyse the patient's progress within the programme in more detail and provide a more tailored plan.

Based on this, this paper presents a real-world long-term study where a socially assistive robot was used to provide motivation and feedback to the patients, aimed to support CR phase II therapies and improve the adherence. This is the first in-depth clinical study that explores the benefits of using a socially assistive robot for long-term cardiac rehabilitation in terms of adherence and physiological progress. Furthermore, in contrast to our previous studies, where we analyzed patients on a case-by-case basis, this paper includes the analysis for the physiological progress through the complete CR programme (36 sessions) for all the patients recruited during the study, in addition to the perceptions of the clinicians that were part of the study for 2.5 years. The remainder of this work is organized as follows. section 2 presents Socially Assistive Robotics (SAR) studies focused on healthcare conditions. section 3 describes the interface architecture used in the CR environment. section 4 presents the protocol and experimental design carried out. section 5 presents the results from the study, section 6 highlights findings and discusses the implications, and section 7 presents the conclusions of the study.

## 2. Related Work

Socially Assistive Robotics (SAR) is a domain of Human-Robot Interaction (HRI) which focuses on developing robots capable of assisting users through social interaction (Feil-Seifer and Matarić, 2005; Matarić and Scassellati, 2016). Unlike virtual agents, socially assistive robots present a physical embodiment, which improves likeability (Fasola and Matarić, 2013; Li, 2015), user engagement and motivation (Vasco et al., 2019), adherence (Bickmore and Picard, 2005; Kidd and Breazeal, 2007) and task performance (Vasco et al., 2019), which are essential in long-term healthcare programmes.

Socially assistive robots have been shown to improve user motivation and engagement in several studies in rehabilitation (Kang et al., 2005; Gockley and Mataric, 2006; Matarić et al., 2007a; Fasola and Matarić, 2010; Fasola and Mataric, 2012; Šabanović et al., 2013; Swift-Spong et al., 2015), in addition to improved adherence (Gadde et al., 2011). Moreover, for repetitive exercise during healthcare programmes, encouraging feedback and continuous monitoring are essential to enhance motivation and task performance (Eriksson et al., 2005; Kang et al., 2005). Most research in rehabilitation has been carried out under laboratory conditions or during short-term interventions, which restrict the applicability of their impact in real-world clinical scenarios for long-term rehabilitation, due to the confounding factors, such as the novelty effect (Gockley et al., 2005) and the adaptation of the technology (Leite et al., 2013; Lane et al., 2016; Riek, 2017).

The only prior study in the literature that evaluates using a socially assistive robot in cardiac rehabilitation is that of Kang et al. (2005). A hands-off physical therapy assistant robot was used in spirometry exercises, which the patients were satisfied with. However, the study only analyzed short-term benefits (one session), with a low number of (5) healthy participants under laboratory conditions, and it did not analyse the physiological progress of the patients. Socially assistive robots were also explored within other similar areas of rehabilitation, such as post-stroke rehabilitation, which showed potential benefits, such as increased willingness to perform prescribed exercises and enthusiastic responses toward the robot (Matarić et al., 2007a).

Perceptions of healthcare staff toward using SAR may be initially negative due to the common concerns or doubts (Winkle et al., 2018; Casas et al., 2019). A common negative perception toward robots is believing them to be potential replacements of their job. However, SAR relies on the collaboration of the medical staff to provide individual support to the patient and help the medical staff in monitoring the patients more closely. Some studies have shown that these perceptions can improve after demonstrating the benefits of using a socially assistive robot (Winkle et al., 2018; Casas et al., 2019). In addition, clinicians' trust may increase with the repeated use of the robot and by positive recommendations of other healthcare professionals in the field (Carrillo et al., 2018). The adoption of the technology by the healthcare staff depends heavily on the system's reliability and their resulting trust (Winkle et al., 2018; Langer et al., 2019). Nonetheless, incorporating social robots into the real-world healthcare programmes poses several challenges, such as technical failures of the robot or sensors, which may negatively affect the perception of the patient or the healthcare staff, and result in a decrease of expectations and engagement (Süssenbach et al., 2014), or generate a false sense of security that can lead to risks.

In our previous work (Lara et al., 2017; Casas et al., 2018c), we described the technical components of a sensor interface for obtaining patients' cardiovascular parameters (e.g., heart rate, recovery heart rate, and blood pressure) and spatiotemporal gait parameters (e.g., cadence, step length, and gait speed), in addition to the exercise intensity parameters (e.g., exertion level and treadmill inclination) and gaze direction indicating the cervical posture. We aimed to investigate the effects of using a socially assistive robot that uses these sensory values to give verbal feedback to the patient, in comparison to a *Control* condition, where the sensory values are only displayed on a tablet-based Graphical User Interface (GUI), i.e., no verbal feedback is given to the patient. Initial user studies (Lara et al., 2017; Casas et al., 2018a,b; Casas et al., 2018c; Casas et al., 2020; Aguirre et al., 2020; Irfan et al., 2020) validated the sensor interface and the robot within the laboratory and clinical settings based on case studies. In Casas et al. (2019), we compared the perceptions of the patients that have conducted the cardiac rehabilitation programme with the robot to the expectations of the patients that have not interacted with the robot through a control group. The results showed a significant increase in the perceptions of the robot's usefulness, sociability, and safety for the robot condition, in comparison to the expectations of the control group. In addition, patients in the robot condition expressed the desire to interact with the robot again. They also commented on its benefits on their progress during the sessions. Besides, a focus group was formed to evaluate the perceptions of the clinicians on the CR programme supported by the robot. After the initial questionnaire and discussion, the robot's capabilities were presented. The demonstration improved the perceptions of the clinicians for the robot's usefulness and safety, and facilitated trust to the system. The clinicians found the continuous monitoring aspect of the robot to be helpful in the case of emergencies and for applying high-intensity parameters during the training, in addition to helping them focus more on the needs of the other patients. Consequently, in this work, we present the perception of the clinicians who have worked with the robot and the findings based on the patients' physiological progress and interactions with the robot.

## 3. Patient-Robot Interface Description

To evaluate the effect of a socially assistive robot in cardiac rehabilitation, this study compares two conditions: *Control* and *Robot*. We used a NAO robot from Softbank Europe^2^, a social robot widely used in human-robot interaction research. A patient-robot interface based on two modules was developed, as previously described in Lara et al. (2017). The first module includes the *sensor interface* that allows physiological parameters monitoring, and the second module, the *robot module* is in charge of the interaction and the social component, i.e., motivation and immediate feedback throughout the exercise.

### 3.1. Sensor Interface

**Figure 1:**
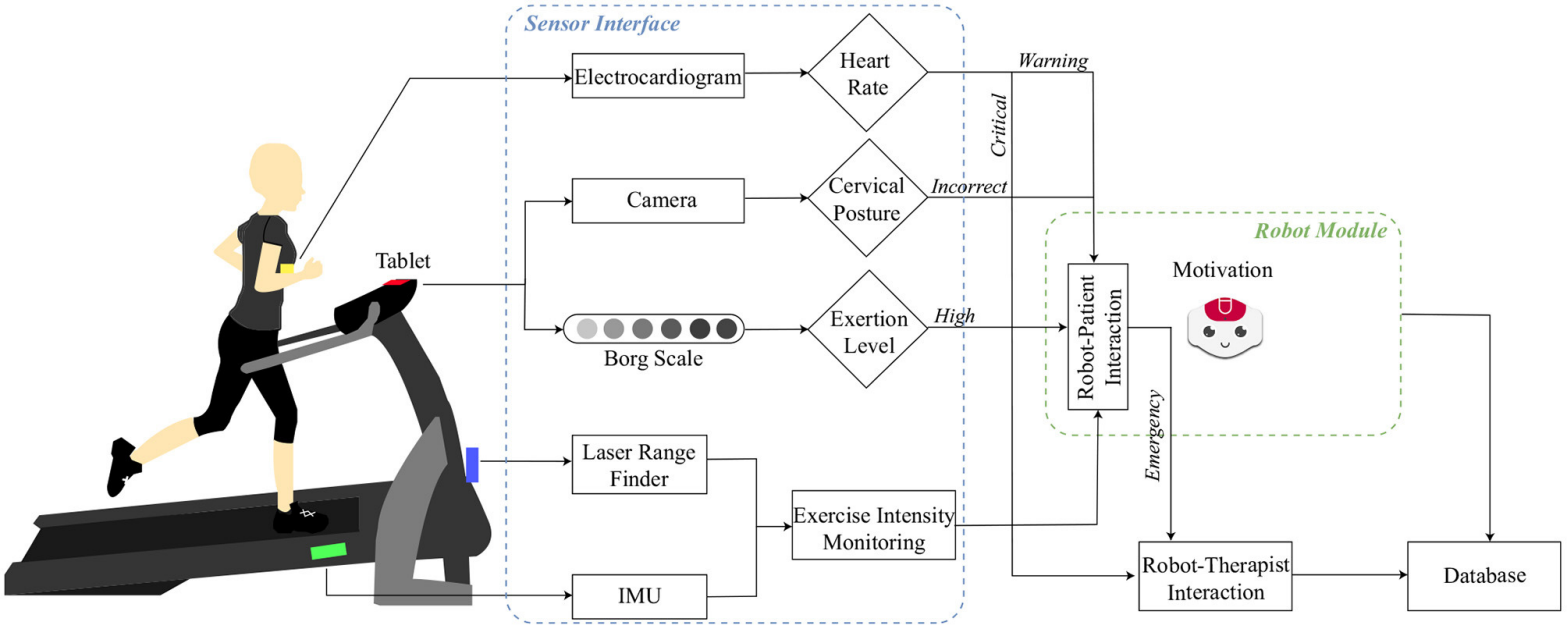
A diagram of the patient-robot interface used in the cardiac rehabilitation programme.

As shown in Figure 1, a set of sensors were integrated to measure each parameter. The details of each parameter are explained in this section.

#### 3.1.1. Cardiovascular Parameters

In cardiac physiology, several physical parameters are useful for studying the activity and regulation of the heart (Aamot et al., 2014). In CR, there is an increased interest to measure these parameters as they reflect the performance and progression of the patient. Consequently, our study analyses these parameters at different phases of the exercise:

- *Training Heart Rate (THR)* [bpm]: The average heart rate obtained during the treadmill exercise (15–20 min). The heart rate values were measured using an electrocardiogram Zephyr HxM sensor^3^ placed on the patient's chest.
- *Recovery Heart Rate (RHR)* [bpm/bpm]: This value represents the difference between the patient's *THR* and the average heart rate 1-min post-training (average of 60 values^4^) (Equation 1):

(1) $$\begin{array}{r} RHR=THR-HR_{post-training} \end{array}$$

To reduce the subjectivity of the measurements that change between the patients and increase the homogeneity, the *RHR* is normalized with the initial resting heart rate (*IHR*), which was taken by the clinicians when the patient arrives in the clinic (Equation 2).

(2) $$\begin{array}{r} RHR_{normalized}=RHR/IHR \end{array}$$

#### 3.1.2. Gait Spatiotemporal Parameters

Healthy gait is described as a series of rhythmical, alternating movements of the trunk and limbs, which results in the progression of the center of gravity and the body (AposTherapy, 2021). The patient's gait performance is analyzed by the gait components, which can be categorized under the following distance measurements (spatial) and time (temporal parameters):

- *Cadence* [steps/min]: The total number of full cycles taken within a given period (Thompson, 2002).
- *Step Length* [m]: The distance between the point of initial contact of one foot and the initial contact of the opposite foot (Thompson, 2002).
- *Gait Speed* [mph]: This variable refers to the normal walking speed adopted by a person in everyday life (Thompson, 2002). Also, this variable represents the treadmill's speed.

In this case, a Hokuyo-URG 04LX-UG01^5^ Laser Range Finder (LRF) was used to acquire these parameters during the session.

#### 3.1.3. Cervical Posture

Cervical posture corresponds to the flexion of lower cervical vertebrae and their inclination (Shafer, 1987), which corresponds to the head inclination in sagittal, coronal and transverse planes. To measure this parameter the front camera of the tablet^6^ placed on the treadmill screen; and a head gaze estimator (Lemaignan et al., 2016) were used. During the exercise, a correct cervical posture is achieved when the patient looks straight ahead (i.e., the head gaze vector's vertical component is above 5.73 degrees). As the CR sessions are performed on a treadmill, the proper posture is essential to avoid dizziness, falls and nausea (Martin and McConahay, 1972).

#### 3.1.4. Physical Activity Intensity Parameters

**Figure 2:**
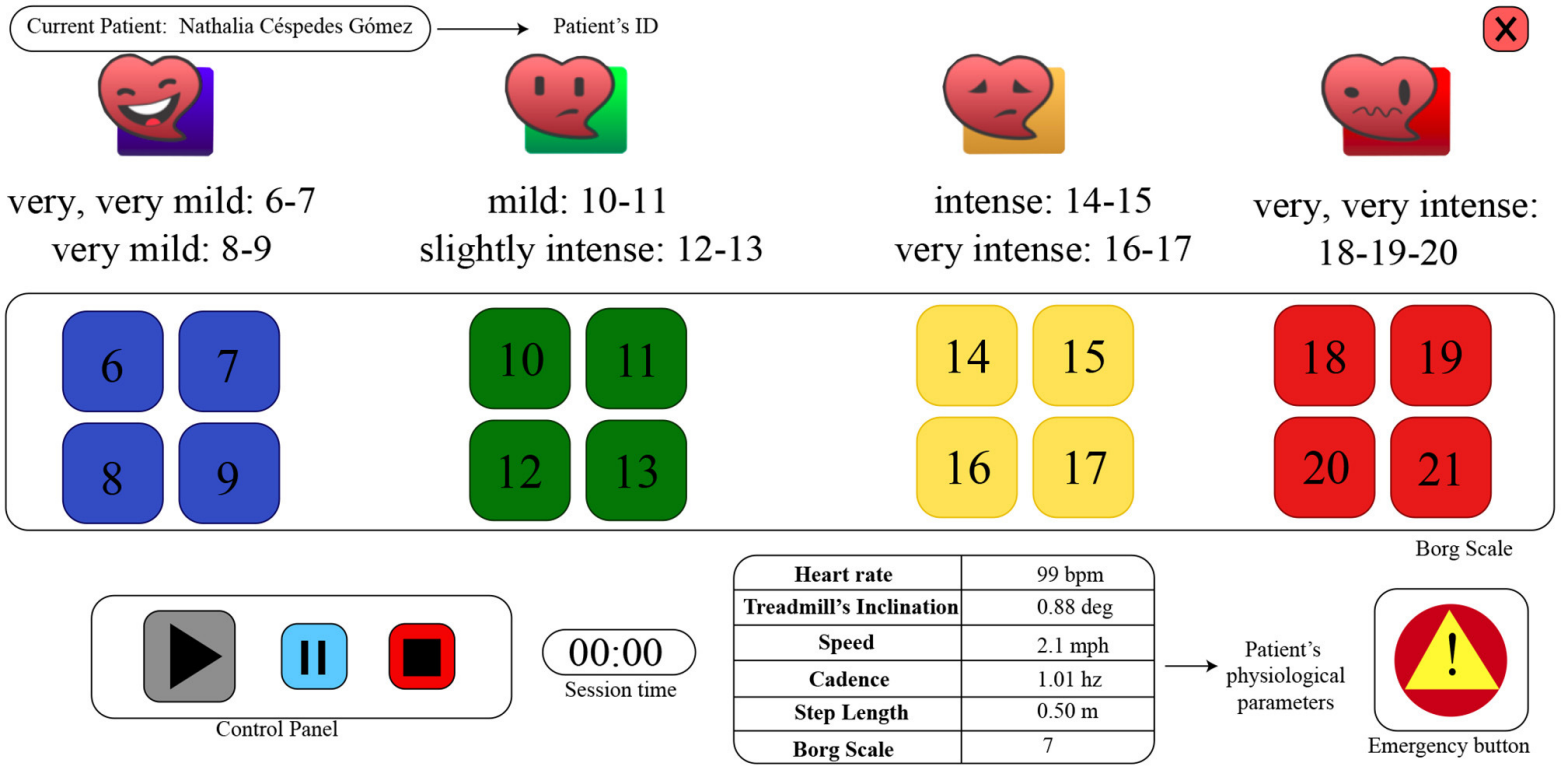
The Graphical User Interface (GUI) of the tablet mounted on the treadmill. The perceived exertion level of the patient is obtained through the Borg Scale (range 6–21). The patient's physiological and spatiotemporal measures are displayed. The control panel is used to start and interrupt the session, and the emergency button can be used by the patient to notify the medical staff.

Three indicators were used to measure the intensity of the exercise: (1) The inclination of the treadmill, (2) the perceived exertion of the patient, and (3) the treadmill's speed^7^. An Inertial Measurement Unit MPU9150^8^ was placed on the treadmill to measure the inclination, in a range of 0 and 5 degrees angle. The perceived exertion was measured using the Borg Scale (Borg, 1998). The Borg Scale is a subjective measurement commonly used in cardiac rehabilitation (Aamot et al., 2014) that allows the evaluation of the effort and intensity made by a patient during the exercise. At Fundación Cardioinfantil-Instituto de Cardiología the scale is between 6 and 20, where 6 corresponds to a very low level of exertion and 20 corresponds to a very high level of exertion. The safe range is considered to be 6–12 by the medical staff (Figure 2).

#### 3.1.5. Borg Scale Response Time

Borg Scale is periodically (every 7 min) requested through the tablet interface, as shown in Figure 2. For the *Control* condition, an audible signal is given, along with a change of color in the tablet interface (i.e., the Borg scale is grayed out, when inactive), whereas, the robot verbally asked the patients to enter their exertion level on the tablet each time. The *Borg Scale Response time* [s], represents the time taken by the patient to answer a Borg Scale request made by the robot. In the case of the control group, this variable was not measured as the interface did not register the events when the request was activated and responded.

**Figure 3:**
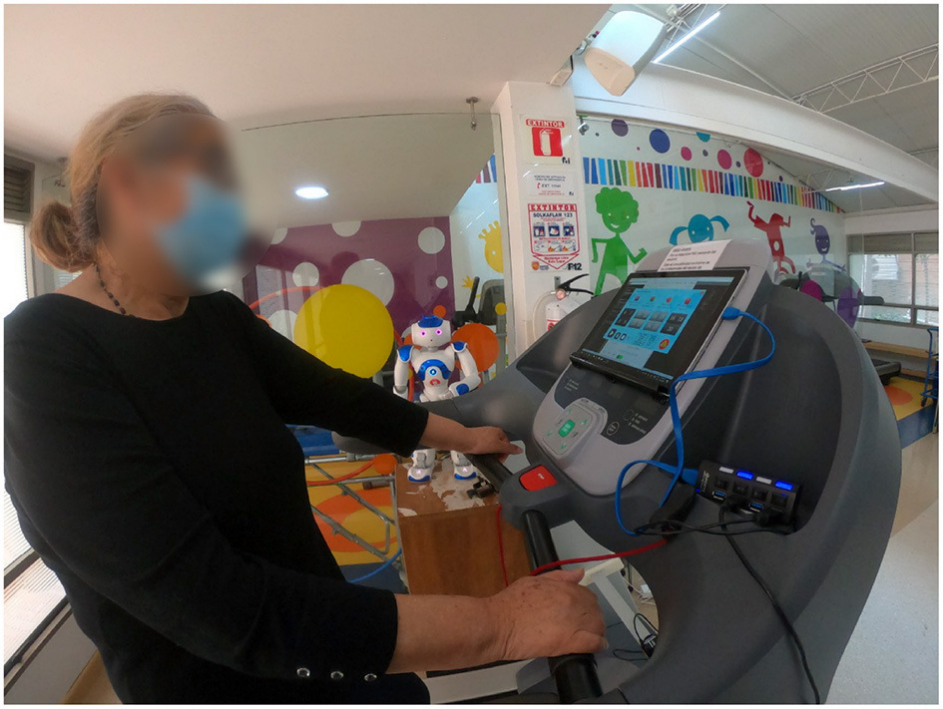
Setup of the experiment with the NAO robot (SoftBank Robotics Europe) and the tablet interface for cardiac rehabilitation at Fundación Cardioinfantil-Instituto de Cardiología clinic.

The patient-robot interface was validated in an initial study under laboratory conditions (Lara et al., 2017). Subsequently, the system was deployed for clinical set-up lasting 18 weeks (36 sessions) at the Fundación Cardioinfantil-Instituto de Cardiología clinic (Figure 3) for 2.5 years.

### 3.2. Robot Module

**Figure 4:**
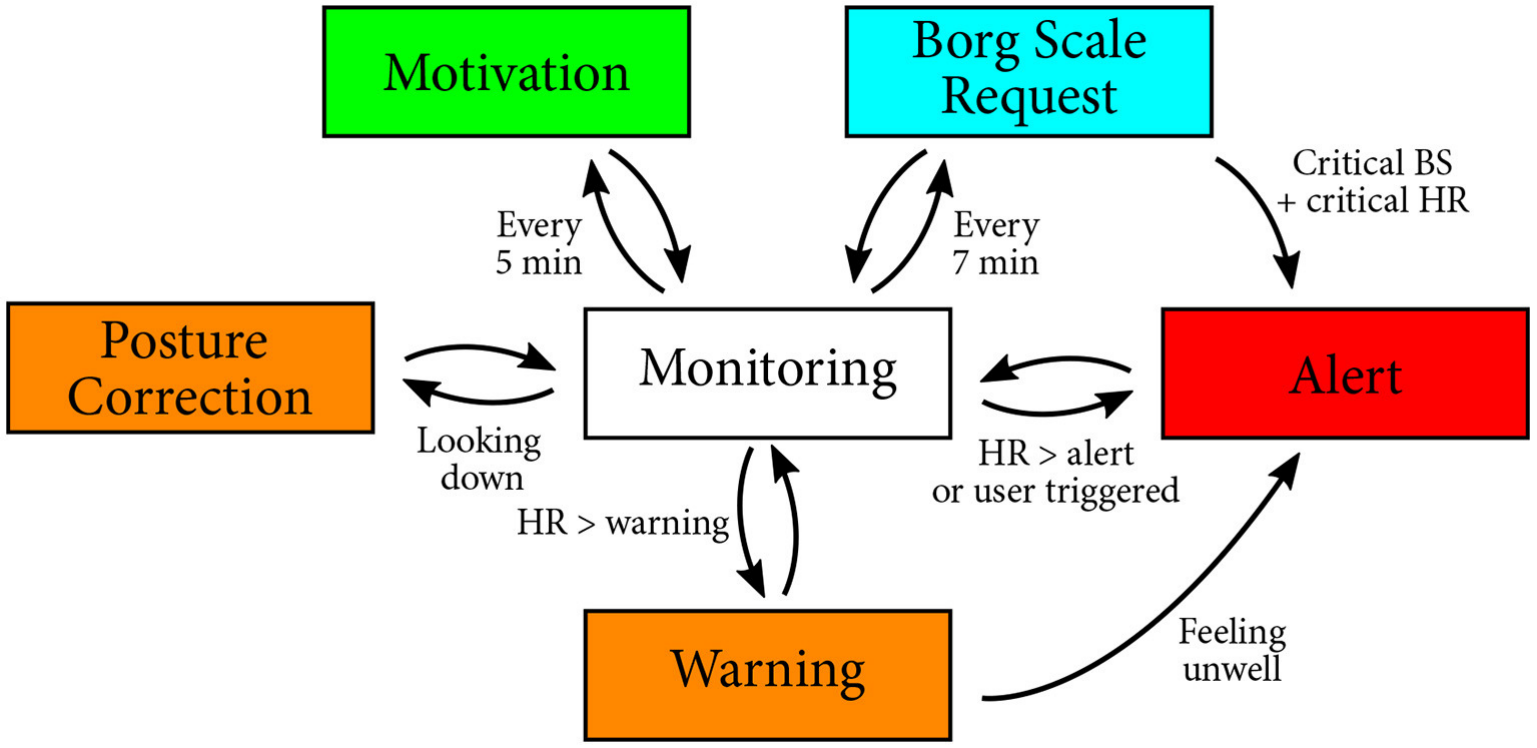
Finite state machine presenting the different transitions possible during the monitoring phase.

The *Robot Module* focuses on interaction with the user. This interaction is divided into three states: (1) *Motivational support*, (2) *Performance monitoring*, and (3) *Online feedback*. A session with the robot starts with an initial greeting, where the robot makes an announcement of the intensity that will be performed during the session (i.e., treadmill inclination and speed). *Performance monitoring* starts when the patient starts their exercise on the treadmill. *Motivational support* occurs periodically every 5 min during the session. The robot encourages the patients through verbal phrases (e.g., “You can do it!”, “You are doing great”” among others), accompanied by non-verbal gestures that are synchronized to verbal speech through the animated speech module^9^ of the NAO robot, in addition to face tracking for gaze maintenance. Similarly, the Borg scale was requested every 7 min to ensure that the exertion level remains within the acceptable range. During this monitoring state, sensory information is analyzed. Depending on the values given by each sensor, the current state can activate the *online feedback* state or remain in the same state. When the *online feedback* is activated, the robot reacts differently based on the event (Casas et al., 2018c): (1) high or critical heart rate, (2) high exertion level, and (3) incorrect cervical posture (See Figure 4). Additionally, we added a cooldown period of 3 min after feedback was provided to prevent the robot from repeating the same feedback too often. When an alert is triggered (i.e., for critical heart rate or by the patient through the tablet interface), the robot calls for help to the medical staff verbally (“Your heart rate is too high, I am calling for help. Doctor, could you please come here?”) with a waving gesture. The alert will be repeated until the medical staff touches the head of the robot to notify their intervention.

#### 3.2.1. Heart Rate Feedback

This feedback is given by the robot when the heart rate exceeds the *warning* or *critical* thresholds. These thresholds are determined before the start of the session by a physiatrist and entered on the tablet GUI. The *warning threshold* corresponds to the maximum of the determined healthy range (i.e., *High HR Warning*). While reaching this value is not critical, it may suggest that the patient is experiencing difficulties with the session intensity. Hence, the robot asks verbally for feedback from the patient through the GUI to report whether they are feeling well. If the patient is not feeling well, the robot alerts the medical staff verbally and non-verbally (i.e., arm gesture). The *critical threshold* corresponds to the maximum heart rate allowed for the patient (i.e., *Call Medical Staff Alert*), calculated by the medical staff using the Karvonen formula (She et al., 2014) (Equation 3), where *HR*_*optimal*_ represents the optimal heart rate during the exercise, *HR*_*max*_ is the maximum HR allowed for the patient, *IHR* means the resting HR, and %*Effort* represents the percentage of desired exercise intensity. Exceeding the *HR*_*optimal*_ level may result in a complication; hence, it is vital to promptly alert the medical staff when this value is reached. Correspondingly, the robot directly alerts the medical staff without confirmation from the patient.

(3) $$\begin{array}{r} HR_{optimal}=\left[\left(HR_{max}-IHR\right)*\%Effort\right]+IHR \end{array}$$

#### 3.2.2. Exertion Level Feedback

This feedback is given when the patient enters the Borg Scale in the tablet. According to the value of the perceived exertion level, three types of robot behaviors are activated during the session: (1) If the Borg scale is on a normal range, the robot thanks the patient, (2) if the patient enters a critical Borg scale (above 12), but the current heart rate is in a healthy range, the robot asks for a confirmation of the Borg scale, and (3) when both the Borg scale and the heart rate are critical, the robot alerts the medical staff.

#### 3.2.3. Cervical Posture Feedback

This feedback is given by the robot when the patient is not looking straight (e.g., looking down at the treadmill or looking sideways). The patient needs to maintain a good cervical posture, i.e., the patient should look straight ahead, to avoid dizziness and other risk factors (e.g., falls). In this case, the robot gives verbal feedback to the patient, asking to maintain a straight posture.

## 4. Methodology

This section describes the protocol carried out to evaluate the effects of a social robot in cardiac rehabilitation. A longitudinal study was conducted lasting 2.5 years for the outpatient phase (II) of cardiac rehabilitation, corresponding to 36 sessions that generally corresponds to 18 weeks if the patient comes twice a week, as prescribed.

### 4.1. Research Question

Our study aims to answer the research question, *What are the benefits of using a socially assistive robot for long-term cardiac rehabilitation?* Based on the previous literature outlined in section 2, we expect an improvement in the motivation, which could improve the adherence to the CR programme and the task performance (i.e., cardiovascular functioning and recovery). Moreover, the loss of interest that may arise in long-term interactions after the novelty effect wears off, can cause a decay in the interaction with the robot and the patient perceptions. Additionally, the collaboration of the medical staff is fundamental in ensuring a rapid intervention in case of emergencies. Thus, their perception of the robot and feedback are central to applications of the robot in clinical scenarios. Correspondingly, in collaboration with the medical staff, we designed a clinical study that incorporates and assesses these elements.

### 4.2. Conditions

To observe the effects of the social robot, two conditions were designed for the study. The details of each condition are described below:

- ***Control condition:*** Within this condition, the participants perform a conventional session of cardiac rehabilitation. As this condition is considered as a baseline, the patients of the *Control* group are monitored only by the sensor interface. In other words, the patient interacts with the tablet only to enter the Borg Scale without the presence of the robot. Therefore, the patients of the *Control* group do not receive any type of feedback or motivation provided by the robot. However, the physiological parameters (i.e., patient's heart rate, gait speed, cadence and step length, treadmill inclination and the previous self-reported Borg scale value) of the patients are displayed on the tablet GUI to inform the medical staff, as shown in Figure 2.
- ***Robot condition:*** This condition integrates the complete described system, that is the patient-robot interface. As shown in Figure 3, the fully-autonomous robot is placed next to the patient below the eye level, to enable the visual attention on the robot's behaviors and feedback. Once the session begins, the robot provides monitoring, feedback and motivation to the patient during the exercise.

The sensor interface and the robot operated fully autonomously. Nonetheless, an experimenter was present in the room during the sessions in both conditions for safety purposes, and only interfered with the session in the case of system failures.

### 4.3. Experimental Criteria

- ***Inclusion criteria:*** The patients considered within this study were those who started phase II (36 sessions) of the cardiac rehabilitation programme that would attend twice a week to the sessions. The patients that are over 25 years old^10^ with acute myocardial infarction (AMI), percutaneous coronary intervention, coronary artery bypass graft (CABG), valve replacement, ischemic heart disease and hypertension and ejection fraction greater than 40% were recruited. Also, these patients have to be able to perform treadmill exercise.
- ***Exclusion criteria:*** Considering the requirements of the system, our system may pose limitations on the patients in the case of any visual, auditive or cognitive impairment that impede the manipulation and correct understanding of the system. Thus, we could not include those patients in the study. The patients that present a different cardiovascular pathology mentioned in the inclusion criteria were also not considered for the experiment.
- ***Dropout and Incomplete criteria:*** Initially, 18 weeks were considered as the CR programme duration, in which patients would attend twice per week. However, some patients missed CR sessions; consequently, this initial policy resulted in a shorter exercise session for the patients (23-33 sessions). Hence, the policy was reviewed in 2018 to improve the CR programme offered to the patients for lasting 36 sessions instead. Thus, a **“drop-out”** is considered when the patient does not attend three sessions in a row, without justification. In this case, the patient is dropped from the study; however, the patient could continue the CR programme without the robot assistance. Furthermore, an **incomplete** CR programme is considered when patients that could not complete the CR programme due to a critical health condition, funding or COVID-19 outbreak, since these reasons are beyond their control.

### 4.4. Participants and Demographic Data

According to the experimental design of the study, we recruited 30 patients (15 per condition). However, as stated in the introduction, the adherence to cardiac rehabilitation is low due to several factors. Consequently, we had 9 patients in the *Control* condition (ages between 44 and 70) and 11 patients in the *Robot* condition (ages between 43 and 80), who actively participated in the rehabilitation and completed the outpatient phase (II) of the cardiac rehabilitation programme. Table 1 shows the demographic data of these patients. The first four sessions of 4 patients (2 in *Control*, 2 in *Robot*) and the complete CR programme of 6 patients (3 in *Control*, 3 in *Robot*) were presented in prior work as case studies (Casas et al., 2018a, 2020). The data for these patients were included in the analysis for this work.

**Table 1:** Demographic data of the patients who have finished the outpatient phase (II) of the CR programme within the study.

	**Control**	**Robot**
Participants	9	11
Gender	9 males	10 males, 1 female
Age (years), mean (SD)	56.6 (7.8)	55.7 (11.2)
Body Mass Index, mean (SD)	26.2 (2.6)	29.2 (3.9)
- Obese	0.0%	54.5%
- Overweight	66.7%	36.4%
- Healthy weight	33.3%	9.1%
**Level of education**		
- Elementary school degree	22.2%	18.2%
- High school degree	22.2%	27.3%
- Technologist	0.0%	18.2%
- Bachelor's studies/degree	55.6%	18.2%
- Postgraduate studies/degree	0.0%	18.2%

The patients' schedules were arranged such that during a CR session with 20 patients, only one subject from the study participated in the session. In other words, 19 other patients present at the CR session were not part of the study, and received conventional CR programme, where the medical staff obtained their measures. While around 200 patients receive CR per day, due to these scheduling restrictions, the availability of the experimenters, and the participation in the study, only 3-5 to patients from the study attended per day.

### 4.5. Measures

To answer our research question, we analyzed the patients' adherence, physiological parameters, how they interacted with the robot and their perceptions of the robot as well as the perceptions of the clinicians that were part of the CR programme with the socially assistive robot.

#### 4.5.1. Dropout Rate

The dropout rate corresponds to the ratio of patients that dropped out based on the withdrawal criteria, to the total number of patients that were recruited in the study.

#### 4.5.2. Physiological Parameters

The main physiological parameters evaluated during the study are the *Cardiovascular* and *Physical activity intensity* parameters (i.e., *RHR, THR*, and Borg scale). A higher *RHR* would suggest an improvement in the health status of the patient, whereas the *THR* and Borg scale would show the difficulties experienced in the sessions.

To analyse the data, six *stages* were proposed. Each stage contains 6 sessions, in total corresponding to the complete CR programme. The analysis per stage was performed to reduce the data and reduce the intrasubject variability.

#### 4.5.3. Interaction With the Robot

Because feedback was not provided in the *Control* condition, the gaze direction of the patient was only estimated in the *Robot* condition. In addition, *Borg Scale Response Time* was only measured for the *Robot* group. Moreover, the counts for the Heart Rate Feedback (i.e., *High HR Warning* and *Call Medical Staff Alert*) were also measured.

#### 4.5.4. Perception of the Robot

As discussed in section 2, using a socially assistive robot for the CR sessions affects not only the patient but also the medical staff. Especially in our study, the collaboration of the medical staff was essential. Hence, the medical staff that interacted with the robot throughout the experiment, which lasted 2.5 years, was interviewed (one physiatrist, one nurse and one physical therapist), to collect their perspectives of the robot and the study. A semi-structured interview was performed online and lasted less than 30 min. Questions regarding their experience (e.g., “*Did you know anything about social robotics before you started the study?”*, “*Were the expectations you had of the robot fulfilled?”*), attitudes (e.g., “*Did you find useful the robot in the programme?”*, “*Which aspects (negative/positive) regarding the robot assistance could you highlight?”*, “*Did the robot change in someway the conventional CR session?”*), and opportunities (e.g., “*Would you continue using the robot?”*, “*If you have the opportunity, which features would you add to the social robot?”*, “*Would you recommend the robot to other healthcare colleagues?”*).

Additionally, an adapted Unified Theory of Acceptance and the Use of Technology (UTAUT) questionnaire (Venkatesh et al., 2003; Casas et al., 2019) was applied to the patients to evaluate their perceptions (or expectations) at the end of their CR programme. The results were previously analyzed in detail in Casas et al. (2019), which showed that the patients perceived the robot positively in terms of ease of use, utility and safety after the completing the CR programme, as briefly outlined in section 2.

### 4.6. Statistical Analysis

The data were analyzed within six *stages* to reduce the intrasubject variability caused by external factors (e.g., illness, tiredness level prior to the session), as suggested by the medical staff. Each stage consists of 6 sessions, in total corresponding to the complete CR programme.

The data for physiological progress, cervical posture and the interactions with the robot are not normally distributed (*p* < 0.00001 in Shapiro–Wilk normality test on residuals and residuals divert from linear reference lines in visual inspection), and the homogeneity of variances assumptions are violated (*p* < 0.05 in Levene's test and/or Box's *M*-test) for training heart rate, inclination and the medical alert. Due to the dropouts and the incomplete CR as previously described, we have unbalanced (non-equal group sizes) data. Moreover, some of the patients completed the CR programme earlier (due to the change in the experimental criteria) and we experienced sensor failures within some of the sessions, hence, we have incomplete (missing) data. Thus, we apply Johansen's (Johansen, 1980) general formulation of Welch (Welch, 1938)-James (James, 1951)'s statistic with Approximate Degrees of Freedom (ADF) (Welch, 1951; Keselman et al., 2003; Villacorta, 2017), which is suitable for non-parametric repeated measures and two-way mixed design (within-subject factor is stages and between-subject factor is condition). The results of the two-way mixed design are reported for stage and condition effects, and their interaction (whether the effect of condition, depends on the stage). We also report the significant differences between conditions for each stage, and the pairwise significant differences between stages for each condition for analyzing longitudinal effects. Hochberg correction for multiple comparisons (pairwise tests) and Least-Squares Estimators (i.e., trimming is not applied on the data) are used, which are the default parameters of the welchADF test in R (Villacorta, 2017)^11^. The results are reported in the format *T*_*WJ*_(*df*_1_, *df*_2_) for the Welch-James ADF test statistic, where *df*_1_ and *df*_2_ are the approximate degrees of freedom for the numerator and denominator, in addition to the *p*-values. The effect sizes are reported based on Glass's δ (Glass et al., 1981; Keselman et al., 2003; Villacorta, 2017) for pairwise comparisons. Note that in contrast to Cohen (1988) and Glass et al. (1981) are critical for classifying effect sizes as “small,” “medium,” and “large,” as the practical importance of an effect depends on the context (i.e., relative costs and benefits) and even small effect sizes can make a substantial difference. Thus, the effect sizes should instead be used to evaluate the consistency and the magnitude of a particular phenomenon across different studies in the literature. Negative effect size denotes that the mean in group 2 (e.g., *Robot condition* or stage 2) is lower than mean in group 1 (e.g., *Control condition* or stage 1), whereas, positive effect size denotes that the mean is higher. Moreover, Chi-Square *Z*-test is applied on recovery heart rate (*RHR*) to determine whether the observed frequencies markedly differ from the frequencies that we would expect by chance.

## 5. Results

Our study aims to improve adherence rate with the use of a socially assistive robot. Correspondingly, we report the patient attendance in section 5.1. In section 5.2, we evaluate the effects of SAR on the physiological progress of the patients during the CR programme of 36 sessions, which was inspected in six stages (6 sessions each). Subsequently, we evaluate how the patients interacted with the robot (section 5.3), and the perception of the patients and the medical staff (section 5.4).

### 5.1. Adherence

**Figure 5:**
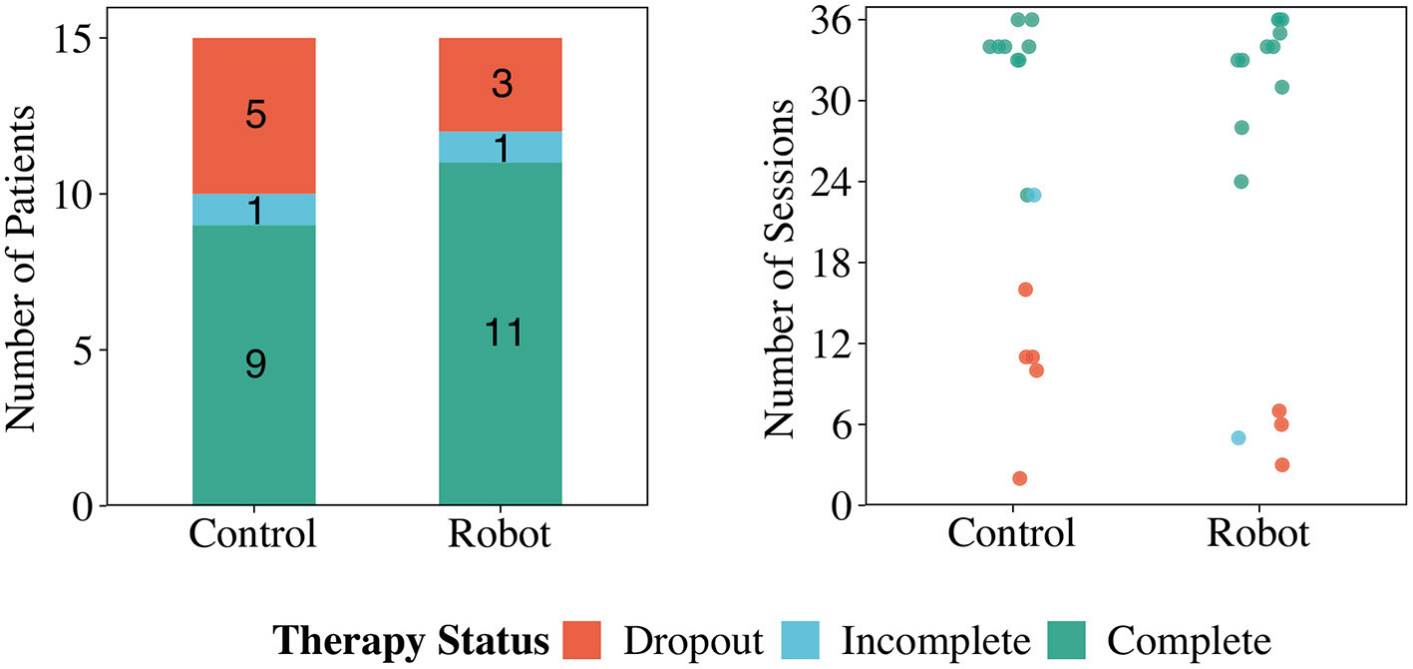
The CR programme status of the users in the *Control* and *Robot* conditions: *complete* refers to the completed cardiac rehabilitation programme as determined by the clinicians; *incomplete* is when patients need to stop the session due to reasons beyond their control, and *dropout* refers to dropping out of the study or not attending three sessions in a row without a justification.

Thirty patients were included in the 2.5 years of the clinical study, corresponding to 670 sessions. However, only 20 patients (Table 1) actively participated in the rehabilitation and completed the outpatient phase as established by the criteria in section 4.3. Figure 5 shows the adherence between the conditions. According to the results, six patients who were in the *Control* condition and four patients of the *Robot* condition did not complete phase II of CR. As it can be observed, these patients attended 11 sessions on average before withdrawing from the CR programme. The dropout rate between the conditions (33.3% for *Control*, 20% for *Robot* condition) shows that the adherence was lower in the *Control* condition than in the *Robot* condition. This outcome was highlighted during the clinicians' interview, where the physiatrist in charge of the rehabilitation expressed that the patients during conventional CR programme generally take more time to finish the CR. To obtain an unbiased (i.e., not affected by participation in a study) baseline adherence rate, we analyzed the medical records in the Fundación Cardioinfantil-Instituto de Cardiología clinic for 14 CR patients that were not part of the study, which showed that patients take 5.7 months on average to finish the outpatient phase (II). In the *Control* condition, this rate was about 4.7 months on average, indicating that patients were committed to completing the programme due their participation in the study. Within the *Robot* condition, this rate was lower, lasting 4.6 months on average. This initial result shows the potential of SAR to improve adherence, which is vital for patients to achieve a complete recovery (Ruano-Ravina et al., 2016).

### 5.2. Physiological Progress

**Figure 6:**
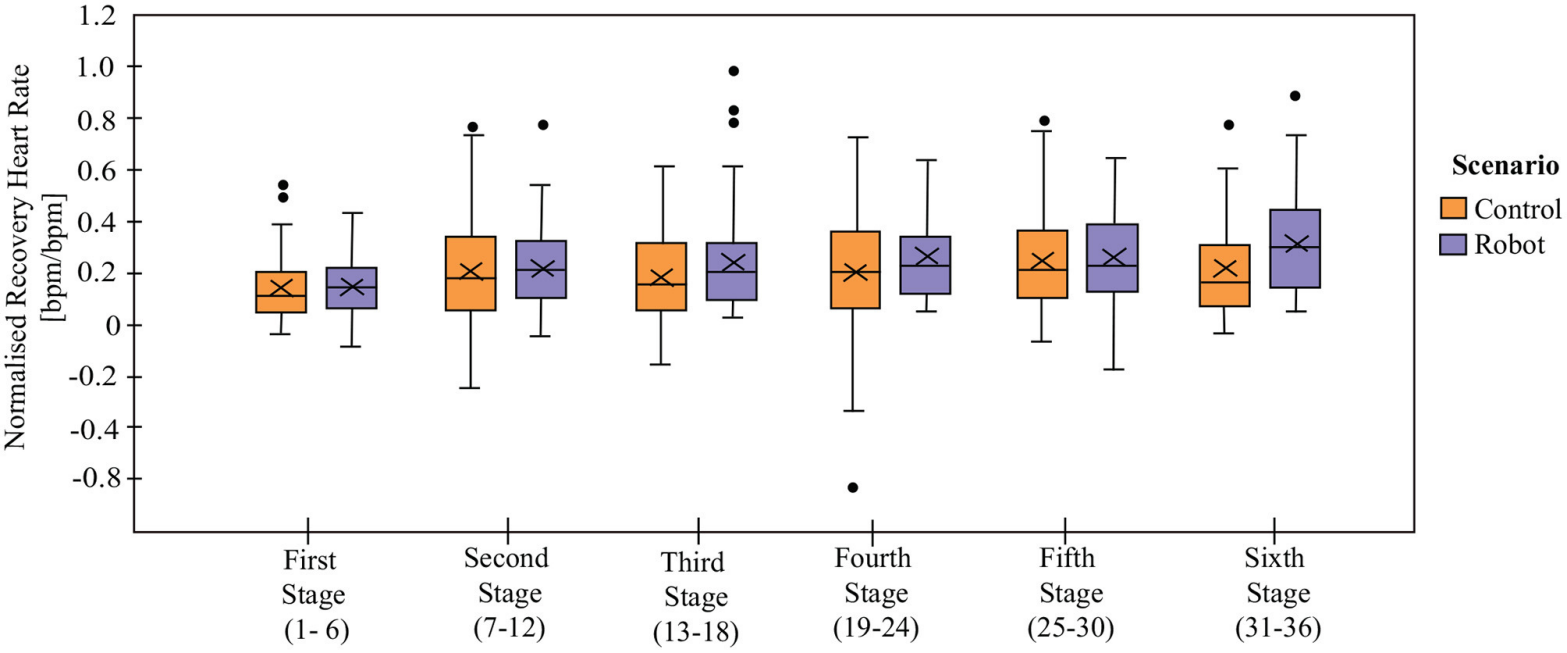
Normalized recovery heart rate (*RHR*_*normalized*_) on each stage in *Control* and *Robot* conditions. Data are normalized with the value of initial resting heart rate on each session. X denotes the mean value per stage.

The recovery heart rate (*RHR*) is considered by the clinicians as the primary physiological parameter, as it determines the CR progress. From a clinical point of view, the recovery of the patient changes as the physical exercise influences the patient's cardiovascular functioning. Hence, it is expected that this value increases along with the rehabilitation programme as this tendency elucidate a healthy recovery. Figure 6 shows the comparison between *RHR* in both conditions. When performing the statistical test over the normalized *RHR*, the results highlight significant differences between stages [*T*_*WJ*_(5, 238) = 11.02, *p* < 0.001] showing the expected behavior of the *RHR* throughout the CR programme (Figure 6). Although, the normalized *RHR*s present significant differences between conditions [*T*_*WJ*_(1, 362) = 12.35, *p* < 0.001], the analysis per stage only show differences for the stage 3 (*p* = 0.02, δ = 0.47) and stage 6 (*p* = 0.01, δ = 0.64). No significant differences were found for the “interaction” between conditions and stages [*T*_*WJ*_(5, 238) = 1.39, *p* = 0.23]. Moreover, Table 2 shows the results regarding the differences between stages within the same condition. As it can be observed, the *Robot* condition exhibits significant differences compared to the initial stage after stage 3 with greater increments than the *Control* condition. This result may arise from the decrease of recovery heart rate (*RHR*) of two patients in the *Control* condition. No other significant differences were observed between consecutive stages, which suggests that the CR programme has rapid positive effects on patient recovery.

**Table 2:** Welch-James ADF results (p-value and effect size in parentheses) for normalized Recovery Heart Rate (*RHR*_*normalized*_).

	**Control**	**Increment %**	**Robot**	**Increment %**
Stage 1/Stage 2	*p* = 0.27 *(0.47)*	49.35	*p* = 0.052 *(0.54)*	54.89
Stage 1/Stage 3	*p*= 0.72 *(0.37)*	34.24	***p* = 0.002 *(0.75)***	82.43
Stage 1/Stage 4	*p* = 0.11 *(0.55)*	48.55	***p* < 0.001 *(0.99)***	98.96
Stage 1/Stage 5	***p* = 0.01 *(0.75)***	92.44	***p* < 0.001 *(0.90)***	96.93
Stage 1/Stage 6	*p* = 0.85 *(0.40)*	68.24	***p* < 0.001 *(1.14)***	135.82

*Significant differences (p < 0.05) are highlighted in bold. Consecutive stages and other pairwise comparisons do not exhibit any significant differences*.

According to the literature, an *RHR* greater than 22 bpm represents a healthy value and a successful rehabilitation process (Carnethon et al., 2015). Thus, a Chi-Square *Z*-test was applied to observe the clinical relevance of the *RHR* between the groups. The results show that there exists a significant difference between the *Control* and *Robot* groups [*z*_(1, *N* = 114)_ = −1.82, *p* = 0.03]. As can be seen in Table 3, a higher number of patients exceeded the threshold in the *Robot* group, showing that the patients in the *Robot* condition have a better physical activity performance.

**Table 3:** Chi-square *Z*-test for the Recovery Heart Rate (*RHR*) between *Control* and *Robot* conditions.

	**Frequency per condition**	
	**Control**	**Robot**
*RHR* > 22	9	20
*RHR* < 22	43	42
Total	52	62

*z(1, N = 114) = −1.82, p = 0.033*.

**Figure 7:**
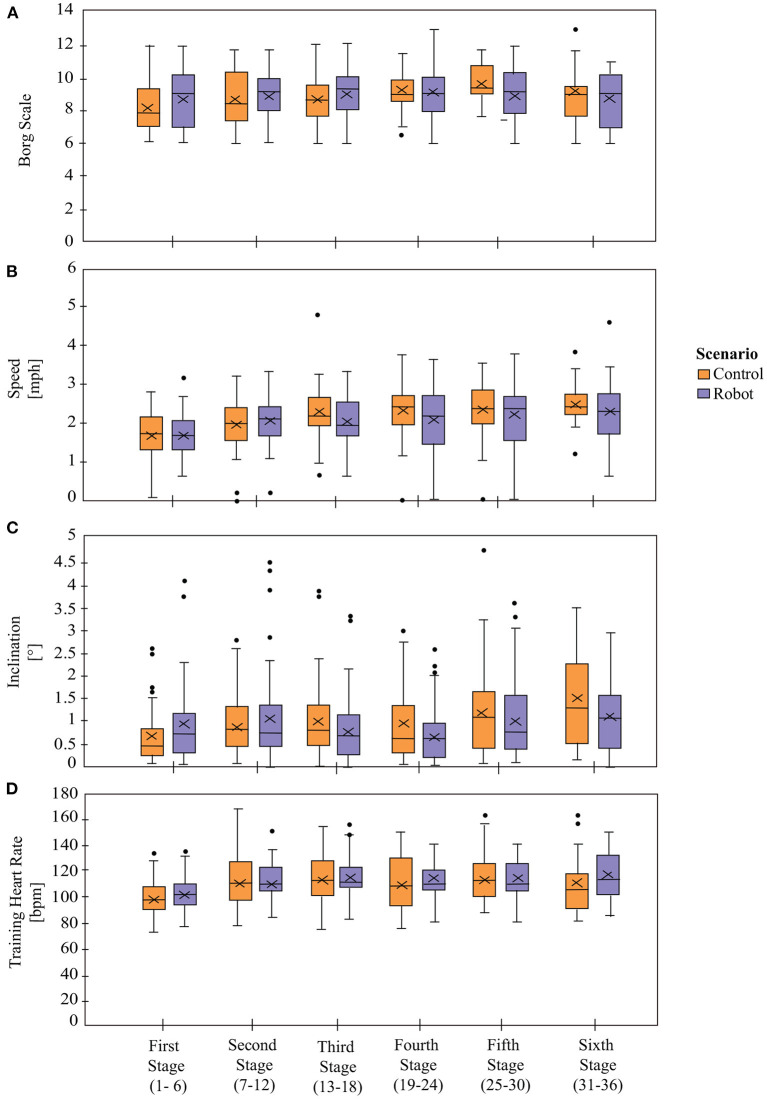
Physiological parameters between *Control* and *Robot* conditions. Panel **(A)** shows the exertion levels of the patients based on *Borg Scale*. Panels **(B,C)** show the *Gait Speed* and the *Treadmill's Inclination*, respectively. Panel **(D)** represent the Training Heart Rate (*THR*). X denotes the mean value per stage.

As CR progresses it is expected that the patients' physiological performance changes, as well as the physical exercise intensity. For instance, the *Borg Scale* (Figure 7A) presents differences between stages independently of the condition [*T*_*WJ*_(5, 277) = 3.32, *p* = 0.006], demonstrating that patients perceive exertion differently from one stage to another as the exercise intensity is increasing throughout the programme, especially between the initial stage and the later stages (stage 4, *p* = 0.04, δ = 0.79, and stage 5, *p* = 0.004, δ = 1.0). The comparison between the *Control* and the *Robot* conditions did not present significant differences overall [*T*_*WJ*_(1, 512) = 0.28, *p* = 0.59]. However, there exists significant differences between the conditions for stage 5 (*p* = 0.003, δ = 0.59). From the longitudinal perspective (comparison between stages of the same condition), the *Control* group presents significant difference between stages 1 and 4 (*p* = 0.004, δ = 0.73), stages 1 and 5 (*p* < 0.001, δ = 1.12), whereas, none of the stages present significant differences in the *Robot* condition. In agreement with these results, the “interaction” of stage and condition for the *Borg Scale* present significant differences [*T*_*WJ*_(5, 277) = 2.89, *p* = 0.01].

The physical activity parameters that determine the session exercise intensity are the *Gait Speed* (Figure 7B) and the *Treadmill Inclination* (Figure 7C). In both cases, the results show that there are significant differences between stages [*Speed*: *T*_*WJ*_(5, 256) = 14.95, *p* < 0.001 and *Inclination*: *T*_*WJ*_(5, 233) = 4.92, *p* < 0.001], demonstrating that the physical exercise parameters change (increase or decrease) across the duration of the CR programme based on the patient's performance and the physiological progress during the sessions. The difference between conditions did not show significant differences [*Speed*: *T*_*WJ*_(1, 485) = 0.59, *p* = 0.44 and *Inclination* *T*_*WJ*_(1, 381) = 3.46, *p* = 0.06] neither the comparison per stages [*Speed*: *p* > 0.05 and *Inclination*: *p* > 0.05], which means that the physical activity parameters were prescribed homogeneously for both groups. For the comparisons between the stages within the same condition, the *Speed* presents significant differences in both groups as shown in Table 4. The *Inclination* presents no significant differences between stages in the *Robot* condition, whereas there are significant differences in the *Control* condition between stages 1 and 5 (*p* = 0.003, δ = 0.83) and stages 1 and 6 (*p* = 0.004, δ = 0.98).

**Table 4:** Welch-James ADF results (*p*-value and effect size in parentheses) for Gait Speed.

	**Control**	**Increment %**	**Robot**	**Increment %**
Stage 1/Stage 2	*p* = 0.17 *(0.48)*	22.66	*p* = 0.12 *(0.46)*	16.18
Stage 1/Stage 3	***p* < 0.001 *(0.96)***	44.06	***p* = 0.003 *(0.66)***	22.64
Stage 1/Stage 4	***p* = 0.002 *(0.79)***	41.66	***p* = 0.02 *(0.57)***	22.98
Stage 1/Stage 5	***p* < 0.001 *(0.89)***	42.56	***p* = 0.001 *(0.78)***	24.36
Stage 1/Stage 6	***p* < 0.001 *(1.21)***	45.58	***p* = 0.001 *(1.04)***	41.49

*Significant differences (p < 0.05) are highlighted in bold. Consecutive stages and other pairwise comparisons do not exhibit any significant differences*.

In accordance with the previous results, there are significant differences between stages for the *Training Heart Rate* (*THR*) [*T*_*WJ*_(5, 194) = 3.31, *p* = 0.007], which is to be expected as the session exercise intensity (determined by the speed and inclination) is changing throughout the CR programme (Figure 7D). While significant differences are found between the conditions [*T*_*WJ*_(1, 309) = 4.35, *p* = 0.04], there does not exist any significant differences between the conditions per stage (*p* > 0.05). Moreover, there is a lack of significant differences on the interaction of stage and condition [*T*_*WJ*_(5, 194) = 0.76, *p* = 0.58]. When the differences between stages are analyzed separately per condition, the *THR*s in the subsequent stages are found to be significantly different than the initial stage for the *Robot* condition, as presented in Table 5, whereas the stages were found to be significantly equivalent for the *Control* condition.

**Table 5:** Welch-James ADF results (*p*-value and effect size in parentheses) for Training Heart Rate (*THR*).

	**Control**	**Increment %**	**Robot**	**Increment %**
Stage 1/Stage 2	*p* = 0.41 *(0.44)*	9.48	***p* = 0.03 *(0.55)***	8.72
Stage 1/Stage 3	*p* = 0.71 *(0.39)*	8.59	***p* = 0.002 *(0.70)***	11.47
Stage 1/Stage 4	*p* = 0.59 *(0.41)*	8.92	***p* = 0.006 *(0.64)***	10.47
Stage 1/Stage 5	*p* = 0.21 *(0.52)*	12.53	***p* = 0.01 *(0.63)***	10.71
Stage 1/Stage 6	*p* = 0.99 *(0.16)*	6.18	***p* = 0.01 *(0.77)***	13.21

*Significant differences (p < 0.05) are highlighted in bold. Consecutive stages and other pairwise comparisons do not exhibit any significant differences*.

### 5.3. Interaction With the Robot

**Figure 8:**
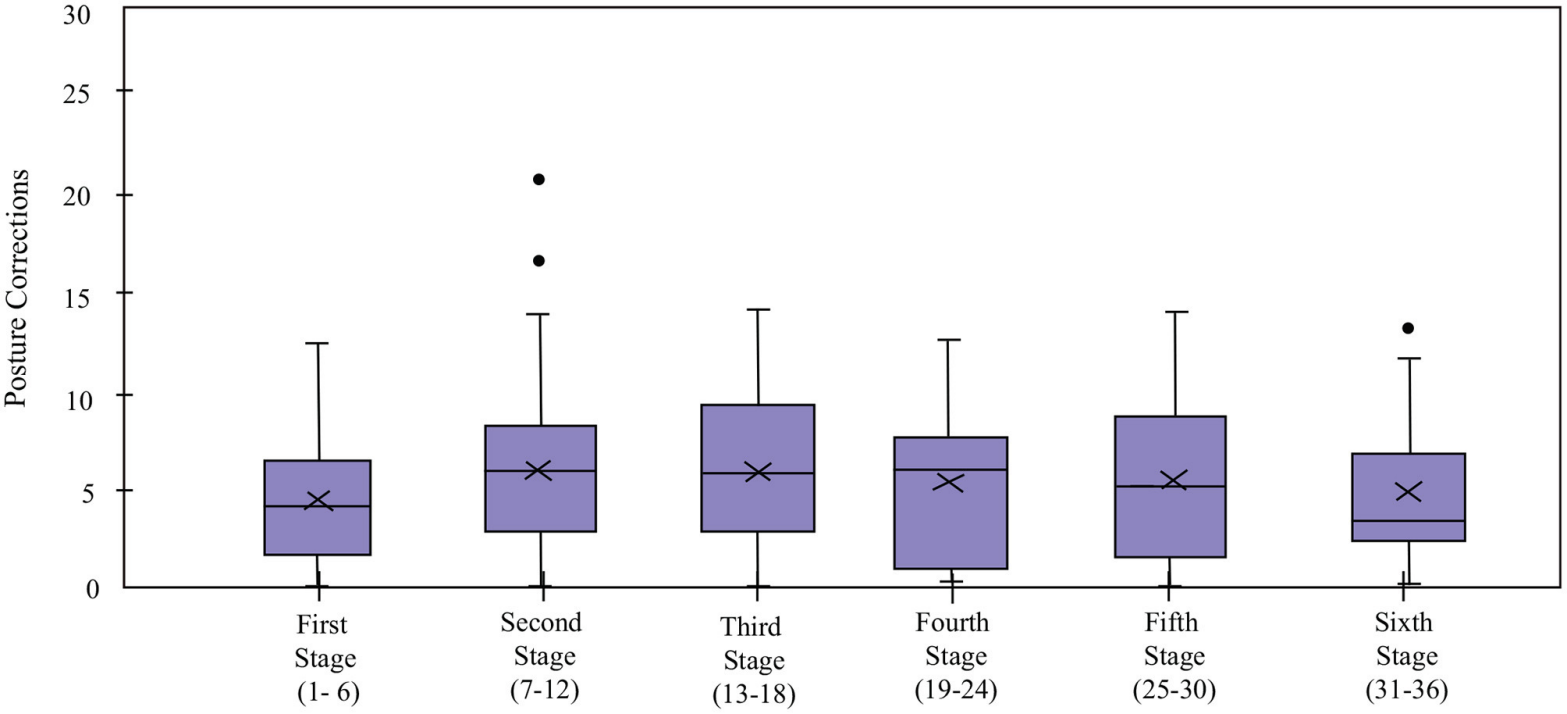
*Cervical posture correction* count of the patients in the *Robot* condition in the cardiac rehabilitation programme. X denotes the mean value per stage.

**Figure 9:**
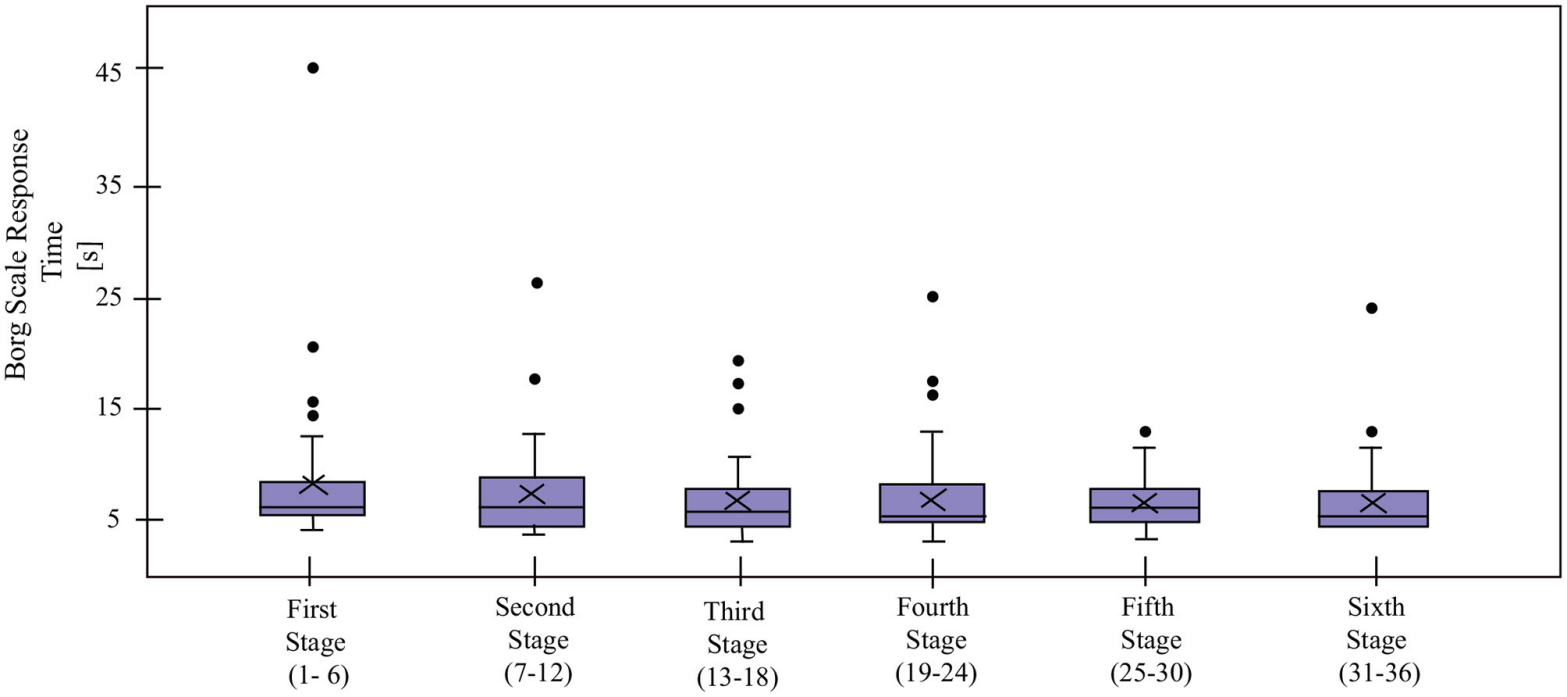
*Borg Scale Response Time* for the patients in the *Robot* condition in the cardiac rehabilitation programme. X denotes the mean value per stage.

**Figure 10:**
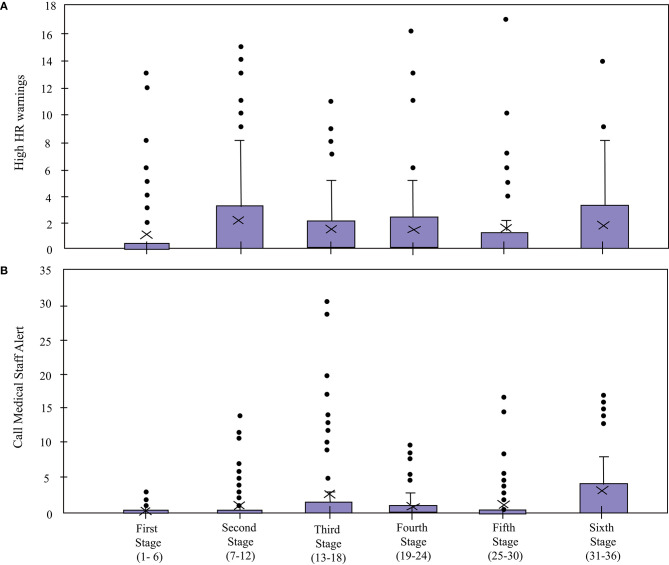
*High HR Warning* and *Call Medical Staff Alert* count of the patients in the Robot condition in the cardiac rehabilitation programme. X denotes the mean value per stage.

Regarding the interaction with the robot, four indicators were measured for the *Robot* condition: *Cervical Posture Feedback* (Figure 8), *Borg Scale Response Time* (Figure 9), *High HR Warning* and *Call Medical Staff Alert* (Figure 10). The results obtained after the statistical analysis elucidate that there are no significant differences between stages for most of the interaction indicators [*Borg Scale Response Time*: *T*_*WJ*_(5, 149) = 1.17, *p* = 0.33, *Cervical Posture Feedback* : *T*_*WJ*_(5, 148) = 1.5, *p* = 0.19, and *High HR warning*: *T*_*WJ*_(5, 154) = 1.05, *p* = 0.39]. These results suggest that the patients tend to maintain their interaction behavior throughout the duration of the CR programme. The similar posture corrections between stages indicate that the patients made efforts to keep a healthy posture. Moreover, the results indicate that the patients continued to respond rapidly to the robot's requests throughout the long-term CR programme.

**Figure 11:**
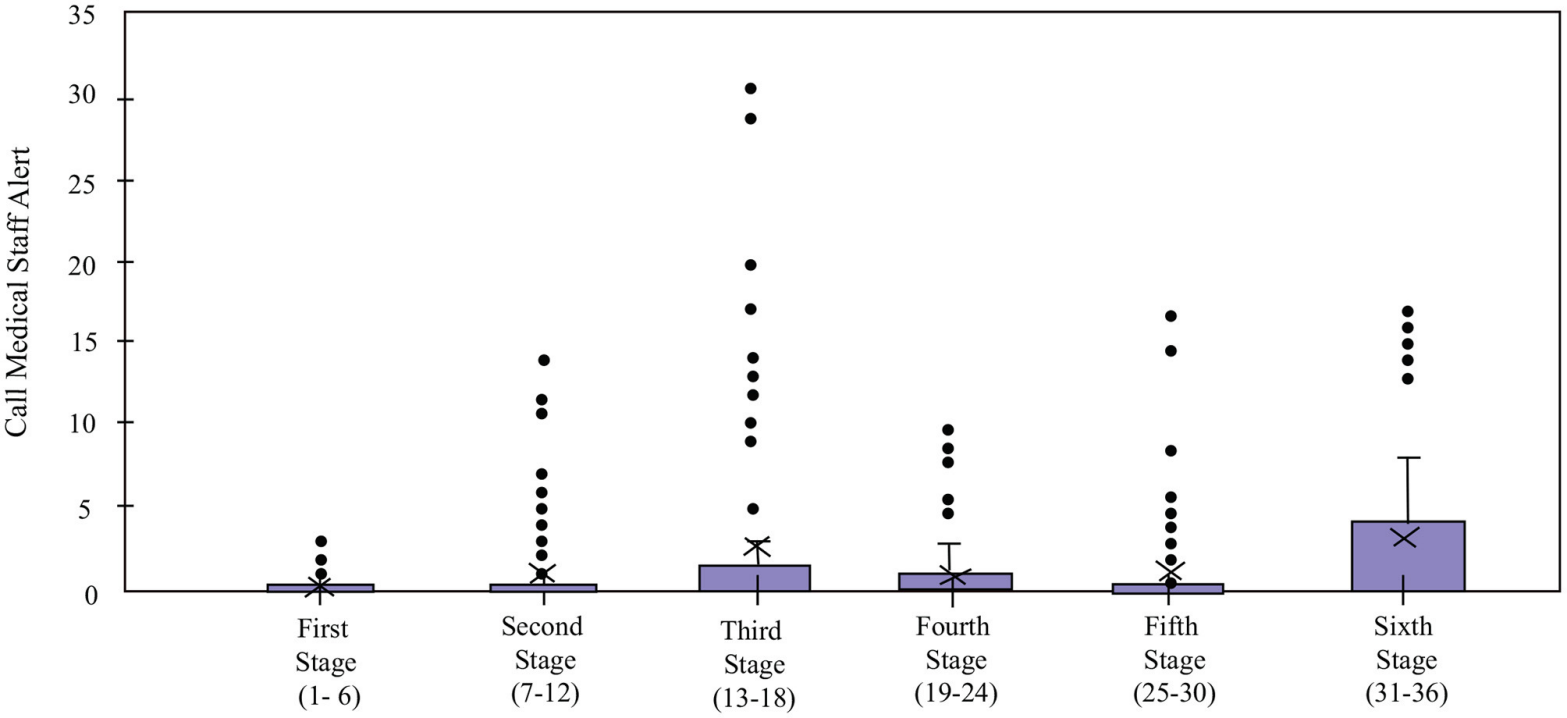
*Call Medical Staff Alert* count of the patients in the *Robot* condition in the cardiac rehabilitation programme. X denotes the mean value per stage.

In contrast, the *Call Medical Staff Alerts* present significant differences between stages [*T*_*WJ*_(5, 132) = 9.67, *p* < 0.001]. As visible in Figure 11, the significant differences per stages were founded between stages 1 and 3 (*p* = 0.02, δ = 0.77), stages 1 and 4 (*p* = 0.005, δ = 0.8), stages 1 and 6 (*p* = 0.02, δ = 1.02). Most of these results are due to sessions where this alert was triggered for a high number of times due to malfunctioning of the robot. While this limitation may have caused false positives, this alarm is very important and vital to the clinicians for immediately detecting an emergency (e.g., symptoms of dizziness or abnormal blood pressure), such that the patient could be supported in a rapid manner (Irfan et al., 2020).

### 5.4. Perception of the Robot

The feedback from the clinicians was positive overall. Most of the clinicians highlighted that the CR felt more secure using the robot due to the continuous monitoring: “*Within conventional sessions our resources are limited, however, the help of the robot enhances the supervision of the session,” “I was more aware of the patient's progress thanks to the robot feedback,”* and “*I feel more secure due to the robot's continuous monitoring”*.

The feedback of the patients during the study included (Casas et al., 2019): “*I feel more compelled to do the exercise because the robot is monitoring me,” “I was very insecure at the beginning of the rehabilitation and thanks to the robot I got confidence,” “I want to come to my rehabilitation, I have the advantage that the robot watches over my health status every second and I feel more secure.”* The clinicians agreed with the positive comments regarding the adherence. When they were asked about the robot's benefits, they commented as: “*The patients who work along with the robot were more engaged with the CR programme,” “The patients who work with the robot were more interested to know their own progress,”* and “*The patients feel more secure with the robot monitoring, and they like the robot, so they have more trust in the rehabilitation.”*

Nonetheless, some of the patients commented on the lack of personalization of the robot: “*I would like the robot to be more personalised,” “I would like the interaction to be closer to the patient (e.g., be more sociable, perform reminders, use patients' name.)”* and “*The robot seems a little repetitive”*.

Finally, the patients and clinicians highlighted that they would like to continue using the robot in the future and they also recommended its use to other patients and healthcare partners during the rehabilitation procedure.

## 6. Discussion

During 2.5 years of the study, 30 patients were recruited to perform CR programme within both conditions. However, only 20 patients completed the treatment. The results include the analysis of 20 patients (9 in the *Control* condition, and 11 in the *Robot* condition) who finished the treatment.

As mentioned in section 1, several rehabilitation programmes present limitations on the adherence to the CR programme. This limitation is caused in part by the psycho-social factors, such as motivation, engagement and anxiety. In this study, the patients' assisted by the social robot had higher adherence to the CR programme, thus, we believe that the robot had a positive effect over the engagement of the patients during the rehabilitation programme due to the continuous monitoring, the feedback and the motivation given to the patients to improve their exercise quality.

Cardiac rehabilitation aims to accelerate recovery and reduce the risk of suffering recurrent events through structured exercises that progressively increase in intensity throughout the programme. The recovery heart rate (*RHR*) is the primary physiological parameter that determines the CR progress. Our results show that the patients assisted by the robot achieved a significantly better recovery overall, obtained a higher increase in *RHR*, exceeded the healthy threshold more frequently, and had a more rapid improvement compared to their initial status, thus, a better cardiovascular capacity functioning. These important clinical results strongly support the use of a socially assistive robot for CR programmes. On the other hand, the results in the *Control* group may be due to the behavior of the *RHR* of two patients, which show a decrease over the rehabilitation procedure.

There were no significant differences in the perceived level of exertion (*Borg Scale*) between the conditions, which was to be expected, as the physical activity intensity parameters (i.e., *Treadmill's inclination* and *Gait Speed*) for both groups were prescribed homogeneously (i.e., no significant differences between the conditions). In contrast, the results exhibit significant differences between stages, demonstrating that the perceived exertion level changes across the duration of the CR programme and depends on the physical activity intensity parameters. For instance, if the *Treadmill's inclination* increases the patient can perceive a higher level of exertion. In particular, for the *Control condition*, the changes were more evident between the initial stage and the later stages (4 and 5) than the *Robot condition* where the comparison between stages did not present differences. This result could suggest that the patients in the *Robot condition* managed to maintain the perceived exertion despite the increase in physical activity, which further support the improvement on their cardiovascular functioning.

Due to the progressively increasing physical activity intensity in the sessions, the training heart rate (*THR*) varies throughout the CR programme. While this change is significant from the initial stage for the patients that were assisted by a robot, no significant differences were found for the *Control* condition. This result correlates with the behavior of the *RHR*, showing that the patients assisted by the robot perform better rehabilitation. These results indicate that the robot has substantial positive effects on the physiological progress of the patients. The underlying reason could be the robot's positive influence on the patients' willingness to achieve the physical activity goals in each session. Furthermore, this outcome can suggest Furthermore, this outcome can suggest that the robot has an influence in the patient's intrinsic motivation, not only through its motivational aspects, but also because of the perceived physiological benefits, which may have improved the patient engagement in the exercise and the CR sessions, because it is personally rewarding.

The monitoring of the physiological data is a critical feature in CR and allow the medical staff to have a better knowledge about the patient's progress. In comparison to the other studies mentioned in the related work section (Kang et al., 2005; Matarić et al., 2007b), this work presents a deeper analysis of the physiological data of patients assisted by a social robot in cardiac rehabilitation.

The continuous monitoring of the *Cervical Posture* by the robot enabled corrective feedback (Figure 8), thereby, reducing the risk of dizziness and falls. The results suggest that the patients managed to maintain their cervical posture throughout the programme.

The *Borg Scale Response Time* (Figure 9) suggests that despite not having prior experience with robots and coming from differing educational backgrounds, all the patients quickly adapted to the technology, and maintained their interaction with the robot throughout the long-term rehabilitation programme. In addition, the experimenters observed that at the beginning of the rehabilitation, the patients take more time to understand the voice and the indications of the robot. Over time, this interaction becomes more fluid due to the experience they acquire. This result shows the importance of the learning curve and how they successfully overcome it. Suggesting an adaptation toward the technology allows long-term interactions and a positive acceptance (Leite et al., 2013). Continuous monitoring of the heart rate enabled providing immediate feedback to alert the medical staff in case of emergencies, which enabled rapid intervention (e.g., through decreasing the physical activity intensity parameters) from them. Hence, despite the fact that the robot malfunctioned and gave an excessive number of alerts on some occasions, this alert proved to be valuable to the medical staff (Irfan et al., 2020).

### 6.1. Potential for Robots in Rehabilitation

This paper presents a real-world scenario for long-term application of a socially assistive robot in cardiac rehabilitation. As stated in section 2, few published studies include a social robot in real-world long-term rehabilitation scenarios and fewer in cardiac rehabilitation. Therefore, this work is a starting point for these applications and the opportunity to improve the quality of life of the patients that suffer from cardiovascular diseases and require cardiac rehabilitation.

While previous work made clear how physically interactive robots could support rehabilitation programmes, our results show that purely socially assistive robots can have a positive impact on CR both on the patients and on the medical staff. First of all, SAR can improve adherence, which prevents up to 50% of the patients worldwide from completing their CR programme (Carlson et al., 2000; Scane et al., 2012), and increase motivation to complete the CR programme faster. Second, we have indications that the social robot could improve cardiovascular functioning, indicating an improvement in the success of the programme. Finally, both the patients and the medical staff reported that they appreciated the robot, acknowledged that it improves the motivation, engagement and trust in the CR programme, and recommended its use for future patients and healthcare staff. The patients perceived the robot as a coaching partner, who help them to carry on their exercise routine throughout the CR programme. In addition, they felt more secure due to the continuous monitoring aspects based on the sensor interface, which allowed them to obtain real-time feedback. As shown in the results, these measurements can decrease risk factors in developing physical activity by giving feedback to the patient and the healthcare staff. This interface could also assess the impact of new strategies with social robots in the patients' physiological state. The patient's adaptation to the robot using interaction based on a touchscreen interface, as shown by the Borg scale response time could be also used in other applications where there is a noisy environment in which the patient may have a difficulty understanding the robot. Important to note is that the medical staff also reported finding the continuous monitoring and immediate feedback of the robot valuable, since the added assurance of the robot's monitoring allows them to focus on the person-to-person interaction with the patient and facilitates rapid intervention in case of emergencies.

### 6.2. Experimental Limitations and Future Work

Nevertheless, our study did have a number of limitations. First, due to the random assignation of patients in the conditions and the relatively limited number of patients, our evaluation had some imbalance between the populations (e.g., unequal gender, obesity, differing education levels). This could be addressed in the future by using larger populations or crossover designs, for example by forming pairs of similar participants and dividing them into different conditions. However, as we recruited participants progressively throughout the study, this method was complex to apply to our study. A second limitation is that our robot's behaviors were identical for each participant, which led some participants to report that they would like the robot to be more personalized. We are currently exploring personalization strategies (Irfan et al., 2020) (e.g., recognizing patients, using their name, and referring to progress in the previous sessions) to address these concerns and improve the adaptation to the robot without the perception of repetitiveness or boredom. Moreover, the patients in the *Control* condition did not receive motivational or physiological verbal feedback to emulate the conventional CR sessions. While most of the physiological parameters were displayed on the tablet interface for the convenience of the medical staff, the lack of any feedback for cervical posture may have affected the results. The robustness of the interface was another limitation presented during the study, as the heart rate sensor was disconnected on some occasions and there was a loss in data. This limitation is being addressed with the development of a new software architecture based on modules to avoid the problems in the acquisition of the sensors. Finally, limitations caused by the malfunctioning of the fully autonomous robot platform and sensors were also present, such as the high number of alerts triggered for the High HR values and some failures on the medical sensors because they were constantly in use for long periods. This last limitation in the sensors was addressed with the acquisition of a new set of sensors.

Other future work could include the interaction with the social robot in different stages of the CR, like warming up and cool down. This interaction could also continue in the patients' homes through virtual agents that could reinforce the healthy habits taught in the CR programme. Concerning the robot system, it could be possible to test other robot platforms like Pepper that could move around in the rehabilitation center and do follow-ups in several patients simultaneously. Finally, integrating other relevant patient measurements would help better understand the patients' performance and guide their CR progress adequately. In this sense, approaches focused on fatigue estimation extracted from cameras and wearable sensors (Pinto et al., 2020) could promote motivation when there are low levels of fatigue, and warning when there are overtraining situations.

## 7. Conclusions

This paper presented the integration of a social robot into the outpatient phase (II) of a cardiac rehabilitation programme. The primary role of the social robot was to assist patients throughout the sessions using various kinds of feedback and interactions. To assess the effect of the robot on the rehabilitation outcomes, two conditions were designed: (i) a *Control* condition, where the patient is enrolled in a conventional CR programme, and (ii) a *Robot* condition, where the patient is assisted and supported by the social robot. A total of 30 patients (15 patients in the *Control* condition and 15 in the *Robot* condition) were included in the study.

However, only 20 patients completed the outpatient phase (9 *Control* condition patients and 11 *Robot* condition), due to dropouts and other external factors. Four main positive conclusions can be drawn from this study: (i) the dropout rate was lower in the *Robot* condition and the patients completed 36 sessions of the programme in a shorter time, (ii) physiological outcomes, as measured by the recovery heart rate, were significantly better in the *Robot* group, and the patients more frequently reached an optimal exercise regime, which indicate a greater improvement in their cardiovascular functioning and recovery, (iii) the patients maintained their interaction with the robot throughout the long-term CR programme, (iv) the clinicians and the patients found the robot valuable for improving perceived safety, patient motivation, and adherence, and recommended its use for future patients and healthcare staff. These results showed the potential of a socially assistive robot in cardiac rehabilitation as a tool to improve the conventional sessions.

## Data Availability Statement

The raw data supporting the conclusions of this article will not be made available by the authors as it contains reserved clinical information. Requests to access the datasets should be directed to marcela.munera@escuelaing.edu.co.

## Ethics Statement

The studies involving human participants were reviewed and approved by Fundación Cardioinfantil-Instituto de Cardiología Ethics Committee. The patients/participants provided their written informed consent to participate in this study. Written informed consent was obtained from the individual(s) for the publication of any potentially identifiable images or data included in this article.

## Author Contributions

NC performed the clinical assessment and data processing. NC and BI performed the statistical analysis and led the manuscript writing. BI and ES contributed with the development of the patient-robot interface for cardiac rehabilitation. MR-R and LG managed the experimental protocol and participated in the clinicians questionnaire development. MM developed the experimental protocol. CC proposed and supervised the structure of the paper. MM, CC, ES, and TB were involved in the revisions and corrections of the manuscript. All authors contributed to the article and approved the submitted version.

## Conflict of Interest

The authors declare that the research was conducted in the absence of any commercial or financial relationships that could be construed as a potential conflict of interest. The handling Editor declared a past co-authorship with one of the authors BI.

## Footnotes

^1^World Health Organization on cardiovascular diseases: https://www.who.int/health-topics/cardiovascular-diseases.

^2^https://www.softbankrobotics.com/emea/en/nao.

^3^Medtronic, New Zealand, https://www.zephyranywhere.com/.

^4^The sample frequency of acquisition corresponds to 1 Hz, 60 values represent 1 min data.

^5^Hokuyo, Japan, https://www.hokuyo-aut.jp/.

^6^Microsoft, USA, https://microsoft.com/es-es/surface.

^7^Corresponding to the patient's gait speed explained above.

^8^InvenSense, USA, https://invensense.tdk.com/.

^9^http://doc.aldebaran.com/2-5/naoqi/audio/alanimatedspeech.html

^10^The patient's age was determined by the medical staff due to the common minimum age of the patients who begin the CR programme.

^11^https://cran.r-project.org/web/packages/welchADF/index.html
